# Ultrasonic Method for the Preparation of Organic Porphyrin Nanoparticles †

**DOI:** 10.3390/molecules15010280

**Published:** 2010-01-12

**Authors:** Mohamad Mehdi Kashani-Motlagh, Rahmatollah Rahimi, Marziye Javaheri Kachousangi

**Affiliations:** Department of Chemistry, Iran University of Science and Technology, Narmak, Tehran, Iran; E-Mails: m.kashani@iust.ac.ir (M.M.K-M.); marzieh.javaheri@gmail.com (M.J.K.)

**Keywords:** porphyrin nanoparticles, optical properties, fluorescence

## Abstract

We report the synthesis and optical properties of organic porphyrin nanoparticles with narrow size distribution and good dispersibility. Nanoparticles were produced by a combination of precipitation and sonication, termed the “ultrasonic method”. The resulting [tetrakis(*para*-chlorophenyl)porphyrin]TClPP nanoparticles were stable in solution without precipitation for at least 30 days. No self aggregation of the constituent porphyrin chromophores was observed. The TClPP nanoparticles exhibited interesting optical properties, particulalrly a large bathochromic shift in the absorption spectra.

## 1. Introduction

Many of the unique properties of nanoscaled materials, especially their electronic, photonic and magnetic properties cannot be achieved using the corresponding atomic, molecular, polymeric or macroscopic materials. Porphyrins are representative of photofunctional organics, and they show remarkable photo-, electro- and biochemical properties that contribute to light harvesting [1]. Hence, meso/nano-scaled porphyrin assemblies or particles are expected to be promising candidates for use in photonic devices [2].

To fabricate organic nano-architectures composed of porphyrins, it should be recognized that van der Waals intermolecular and hydrogen-bonding interactions as well as the electrostatic attraction are responsible for the specific electronic/optical properties that are fundamentally different from those of inorganic metals or semiconductors [3].

The formation of nanoscaled colloidal particles of porphyrins can be accomplished by adding water to a solution of a hydrophobic porphyrin in water-miscible organic solvents such as THF, DMSO, DMF, or CH_3_CN with a few percent of a low molecular weight polyethylene glycol (PEG). This mixed solvent approach is an efficient means to make large quantities of nanoparticle colloids (20–500 nm diameter) of a variety of porphyrins [4,5].

There are a variety of possible applications of porphyrin nanoparticles that derive from the photonic properties of both the component molecule and the nanoscaled dimensions of the particle. Many of these nanoparticles, when the porphyrin contains a redox-active transition metal (e.g., Fe,Co.Mn) are more efficient catalysts on a per porphyrin basis than the individual porphyrins adsorbed onto supports. The fluorescence properties of nanoparticles containing the free base or closed-shell metalloporphyrins or the phosphorescence of these or other metalloderivatives such as the Pd(II) and Pt(II) can be exploited for sensors and displays.

In this paper, we report synthesis of porphyrin nanoparticles via a sonication method. There are only a few reports on the synthesis of porphyrin nanoparticles [4,6,7], furthermore, we acknowledge Drain *et al.*, for the strategy of preparation of porphyrin nanoparticles by a mixed solvent method [8]. 

## 2. Results and Discussion 

### 2.1. Structure of porphyrin nanoparticles

The detailed structure inside these nanoparticles remains unknown. Our hypothesis is that the nanoparticles may consist of sub-domains of the macrocycles and solvent/stabilizer-filled voids or channels of unknown size and distribution, so that the number of chromophores per nanoparticle is substantially less. The structural organization of the porphyrins within the nanoparticles likely depends on the specific structure of the macrocycle used because this dictates the intermolecular interactions between the porphyrins, the solvent and the stabilizer.

### 2.2. Optical properties

**Figure 1:**
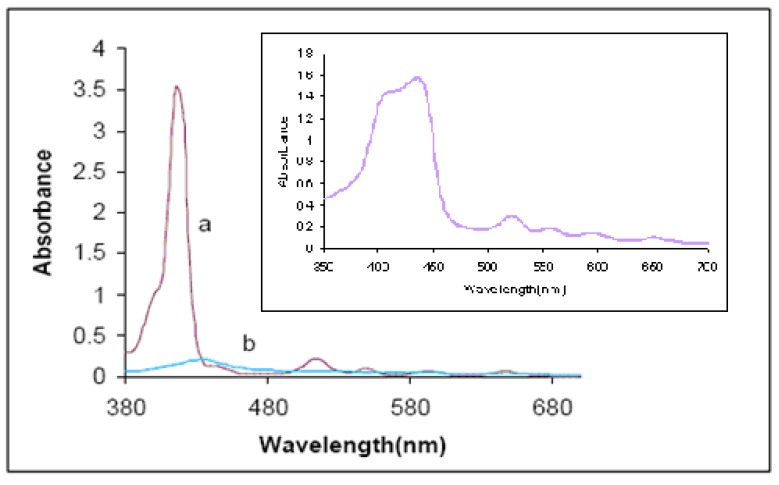
UV-vis spectra of: (a) TClPP in chloroform, (b) nano TClPP; the UV-vis of nanoTClPP is shown at the right.

The UV-vis spectra of porphyrin nanoparticles are significantly different compared to the spectra of the corresponding porphyrin solutions (Figure 1). Soret bands are found to be broadened and/or split. The arrangement of macrocycles in aggregates generally fall into two types, ”J”(edge-to-edge) interactions are characterized by red shifts and “H” (face-to-face) interactions are characterized by blue shifts. The optical spectra suggest both types of interactions in the nanoparticles and are well understood to be indicative of electronic coupling of the chromophores. The extent of J versus H aggregation depends on the specific porphyrin used. The difference between porphyrins in solution and nanoTClPP is clearly shown by the spectra of the nanoTClPP.

### 2.3. Effect of volume of water (guest solvent)

Volume of water did not have major effect on the positions of the Soret and Q bands of the porphyrin nanoparticles, but with increasing volume of water as a guest solvent, the absorbance of the porphyrin nanoparticles decreased. By increasing the volume of the guest solvent, just the solution of the nanoparticles become more diluted and the intensities of the absorption become less but the position of the spectra did not change; this shows that the volume of the guest solvent did not affect the shape of the nanoparticles.

**Figure 2:**
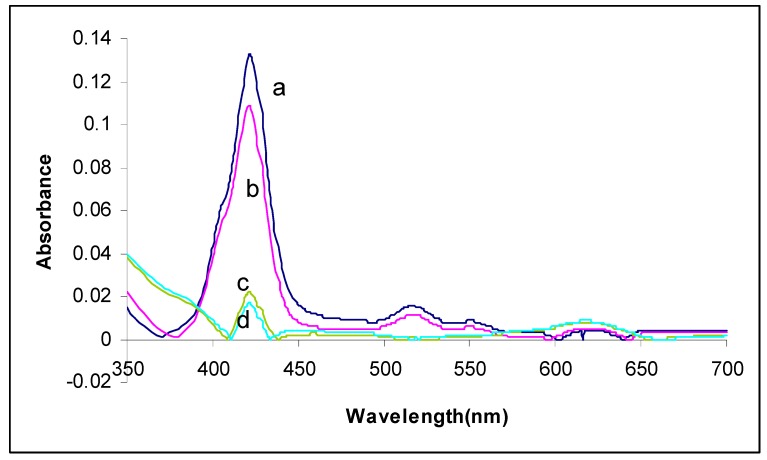
UV-vis spectra: effect of volume of guest solvent: (a): 10 ml; (b) 20 mL; (c) 30 mL; (d) 40 mL.

### 2.4. Effect of concentration of solutions of porphyrin

**Figure 3:**
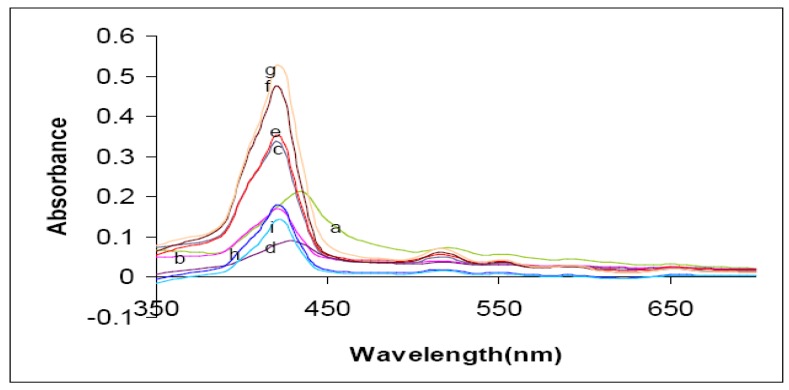
UV-vis spectra: effect of concentration: (a) 200 μL, (b) 250 μL, (c) 300 μL, (d) 350 μL, (e) 400 μL, (f) 450 μL, (g) 500 μL, (h) 150 μL, (i) 100 μL.

The widest range of particle sizes can be achieved by varying the concentration of the macrocycle in the host solvent (Figure 3). The formation of the porphyrin nanoparticles likely represents a process governed by kinetics as well as the equilibrium of intermolecular interactions between all the components. The importance of kinetics is supported by the strong dependence of particle size on concentration in the host solvent—the largest particles form at the lowest concentrations. At the highest porphyrin concentration in the host solvent, there are some differences in the intermolecular interactions of the chromophores since a ~15% broadening of the Soret band is observed, compared to the lowest concentration. The observed larger nanoparticles at lower concentrations may indicate that there is a minimum, critical aggregate size that nucleates the growth, and smaller particles eventually aggregate into larger ones. generally, we can say that at lower concentrations, porphyrins aggregate together and form larger nanoparticles than larger concentrations and because the number of porphyrins per each nanoparticle is more than the higher concentrations of porphyrins, they self-quench their π→π* absorption and because of that the absorption decreases and because the monodispersity of nanoparticles at lower concentrations is more than higher concentrations, the broadening of soret band decreases because monodispersed nanoparticles absorb nearly at the same wavelengths. 

### 2.5. Effect of time of sonication

**Figure 4:**
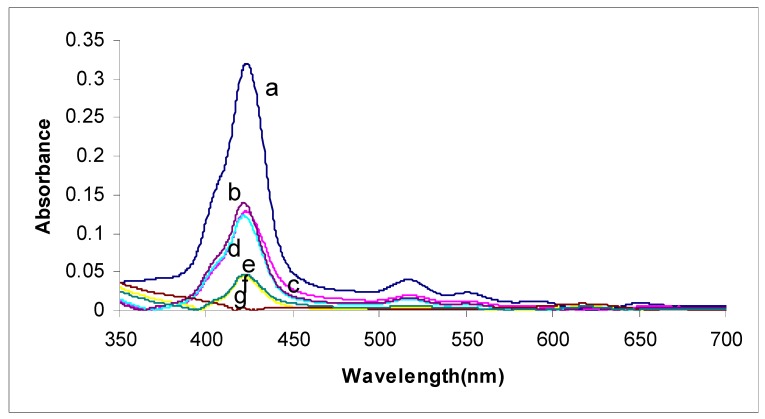
UV-vis spectra time of sonication: (a) 5 min, (b) 10 min, (c) 15 min, (d) 20 min, (e) 25 min, (f) 30 min, (g) 40 min.

Time of ultrasonic process has a profound effect on the particle size of the porphyrin nanoparticles. At less time, as shown in Figure 4, porphyrin nanoparticles have sharper peaks and stronger absorbances; this indicates that by increasing the time of sonication, the number of porphyrin nanoparticles becomes more and the number of porphyrins per each unit of nanoparticle increases.

### 2.6. Mixing

The rate and efficiency of mixing the host and guest solvents have a profound effect on the size and stability of the porphyrinic nanoparticles—especially when metalloporphyrins are used. In general, for a given derivative and using the same rate of addition, the greater the mixing the smaller the nanoparticles. Stirring rates were varied by controlling the spin of a magnetic stirrer and with and without the sonication and sonication assumed to be the most efficient. Smaller and stable porphyrin nanoparticles were formed only with sonication.

### 2.7. Well-dispersed TClPP nanoparticles

**Figure 5:**
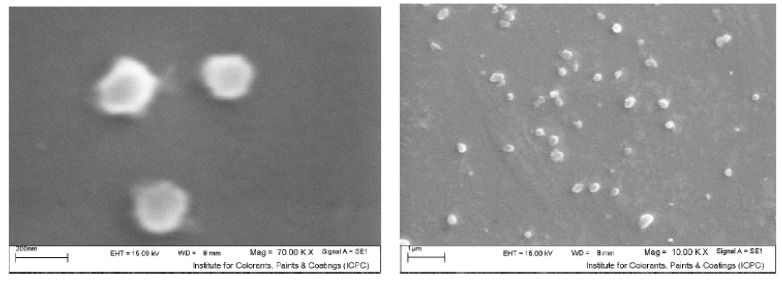
SEM images of porphyrin nanoparticles.

The SEM images of the porphyrin nanoparticles are shown in Figure 5. The particles are spherical in shape and the average size is 200 nm. These nanoparticles were stable in solution without precipitation stored under dark at room temperature for at least 30 days. These images confirm the preparation of porphyrin nanoparticles via ultrasonic method and The porphyrin nanoparticles are held together by hydrophobic and π-stacking effects and hydrogen bonds.

### 2.8. Optical properties of TClPP nanoparticles

**Figure 6:**
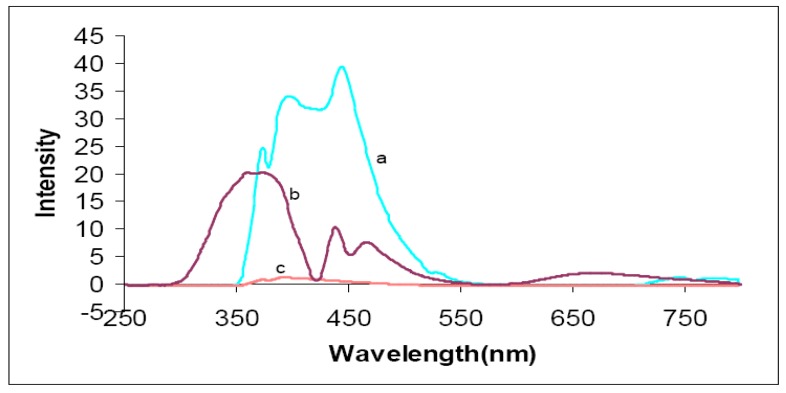
Fluorescence spectra of: (a) TClPP nanoparticle; (b) solution of 0.005 g porphyrin in 20 mL of chloroform; (c) solution of 0.005 g porphyrin in 5 mL of chloroform.

Figure 6 shows a typical fluorescence spectrum of TClPP nanoparticles along with that of aqueous TClPP solution (0.01 mM) excited at 270 nm. The addition of stabilizer neither quenched the fluorescence of solution-phase TClPP nor altered its shape. The fluorescence spectrum of the TClPP nanoparticles exhibited a bathochromic shift compared to that of the solution sample, corresponding to the behavior observed in the absorption. In nanoparticle samples, the Soret band and Q bands exhibit a bathochromic shift compared to those of the aqueous solution. 

Regarding the red shift of the Soret band, four explanations have been proposed in the past [9,10,11]: (i) protonation of the porphyrin ring nitrogens, (ii) solvent (or matrix) effect, (iii) aggregation of porphyrin molecules and (iv) flattening of the porphyrin molecule caused by the twisting of four phenyl moieties. In mechanism (i), a highly acidic solution induces the protonation of the porphyrin ring. The pH of the solutions containing the nanoparticles were measured to be 7.1–7.3. the observation of four Q bands for the nanoparticle samples makes this mechanism implausible because diprotonated porphyrins show only two Q bands due to the *D*_4_h symmetry of the chromophore. In mechanism (ii), although the absorption band maxima depend on the refractive index of solvents or matrices, very minor spectral shifts of the Soret band (~2 nm) have been revealed for the TClPP molecule when the solvents are largely varied. Thus, this mechanism is also not the main reason of the large red shift of the Soret band. In mechanism (iii), two types of molecular aggregates involving dipolar coupling are considered with unique electronic and spectroscopic properties: J- and H-type aggregates. When chromophores such as porphyrins are parallel aligned, two new excitonic bands are generated according to a simple exciton theory: one with higher energies and the other with lower than the monomer energy level. In J aggregates, transitions only to the low energy states of the exciton band are allowed. As a consequence, J aggregates exhibit a red-shifted absorption band with respect to the monomer band, and are characterized by almost no Stokes-shifted fluorescence that has a very high quantum yield. In H aggregates, on the other hand, transitions only to the higher level are allowed, yielding a blue shift of the absorption peak. Another consequence of the dipolar coupling in the H aggregate is quenching of fluorescence caused by a rapid internal conversion to the lower energy level and a subsequent forbidden transition to the ground state, therefore a shift of the Soret band to longer wavelengths should be attributed to the self-association of porphyrins into J-type aggregates. The estimated fluorescence quantum yields of the TClPP nanoparticle systems are quite similar to that of the solution-phase TClPP so that J-type self-aggregation does not occur in the nanoparticles.The origin of the large red shift of the Soret band is then reasonably attributed to a flattening of the porphyrin molecule [mechanism (iv)].

## 3. Experimental Section

### 3.1. Chemicals and instruments

DMF used as a solvent was purchased from Merck and used as received. Triethylene glycol mono-methyl ether was used as a stabilizer and also purchased from Merck. UV–vis absorption spectra were recorded on a Hitachi U-4100 spectrophotometer. Fluorescence spectra were obtained with a Hitachi F-4500 spectrofluorometer. The morphology and size of TClPP nanoparticles were observed with a Hitachi S-4800 scanning electron microscope (SEM).

### 3.2. Synthesis of TPClPP nanoparticle

TClPP was prepared according to the Adler-Longo method [12]. The porphyrin [tetrakis(*para*-chlorophenyl)porphyrin] nanoparticles were prepared by means of a sonication technique. A typical preparation procedure as follows: stabilizer (triethylene glycol monomethyl ether, 100 μL) was added to stock solution of TClPP in DMF (1.22 mM for TClPP, 200 μL), followed by addition of water (20 mL with vigorous mixing and after that was sonicated for 30 min at 60 ºC. In this research, the effect of different variables on the size of nanoparticles was examined via UV-vis spectroscopy [12].

**Scheme 1:**
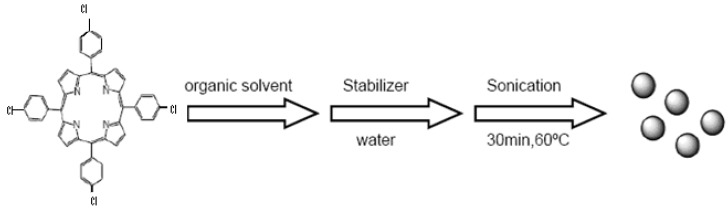
Preparation of porphyrin nanoparticles.

## 4. Conclusions

No self-aggregation of the constituent porphyrins was observed in the nanoparticles. The absorption spectra of nanoparticles exhibited a large bathochromic shift compared to that of the monomer solution. In the fluorescence properties, a spectral resolution increase could be observed compared to that for the corresponding aqueous-phase porphyrin. This method provides a useful way to prepare porphyrin nanoparticles. These nanoparticles can be used as catalytsts for oxidative reactions as well as optical sensors, and we guess that theses compounds act better than their monomers.
